# Supplementary material for: Preserving fairness and diagnostic accuracy in private large-scale AI models for medical imaging
Source: Commun Med (Lond). 2024 Mar 14;4:46. doi: 10.1038/s43856-024-00462-6 (PMC10940659; doi:10.1038/s43856-024-00462-6)
Supplement: Supplementary file 2 — Supplementary Information [file 43856_2024_462_MOESM2_ESM.pdf]

Preserving fairness and diagnostic accuracy in private large-scale AI models for medical imaging - Supplementary Information

Soroosh Tayebi Arasteh[1,+,*], Alexander Ziller[2,3,+,*], Christiane Kuhl[1], Marcus Makowski[2], Sven Nebelung[1], Rickmer Braren[2], Daniel Rueckert[3], Daniel Truhn[1,x,*], and Georgios Kaissis[2,3,4,5,x,*]

[1]Department of Diagnostic and Interventional Radiology, University Hospital RWTH Aachen, Aachen, Germany.
[2]Institute of Diagnostic and Interventional Radiology, Technical University of Munich, Munich, Germany.
[3]Artificial Intelligence in Healthcare and Medicine, Technical University of Munich, Munich, Germany.
[4]Department of Computing, Imperial College London, London, United Kingdom.
[5]Institute for Machine Learning in Biomedical Imaging, Helmholtz-Zentrum Munich, Neuherberg, Germany.
[*]{sarasteh, dtruhn}@ukaachen.de, {alex.ziller, g.kaissis}@tum.de
[+]These authors contributed equally to this work
[x]These authors jointly supervised this work

Supplementary Note 1: Additional remarks on privacy-utility trade-off

Varying model architectures

In addition to the ResNet9-architecture reported in the main manuscript, we additionally used three more architectures: An EfficientNet B0, with 4 017 796 parameters, adhering to the original implementation proposed by Tan et al. [1], with the sole exception of replacing all batch normalization layers with group normalization; DenseNet121, with 6 962 056 parameters, following the original design put forth by Huang et al. [2], again with the exclusive modification of substituting batch normalization layers with group normalization; and ResNet18, with 11 180 616 parameters, following the original blueprint developed by He et al. [3], with the unique alteration of replacing batch normalization layers with group normalization. All three models displayed a trend consistent with the utility penalties we observed for ResNet9 in both DP and non-DP training. Compare also Supplementary Figure 4.

Further datasets

To prevent domain-specific bias in our results, we employed the Artificial Intelligence for Robust Glaucoma Screening (AIROGS) dataset [4]. This dataset comprises 101 354 RGB ocular fundus images from approximately 60 000 patients of diverse ethnicities, aimed at detecting the presence of referable glaucoma. We allocated 80% of the patients—both with and without glaucoma—to the training set, reserving the remaining 20% for the test set. Image pre-processing involved cropping and other schemes as detailed in [5] and [6]. The images were resized to a dimension of $3 \times 224 \times 224$, with 3 representing the number of channels. We adopted the same EfficientNet B0 network architecture, with identical DP and non-DP training parameters as described earlier, with the same $\delta = 6 \cdot 10^{-6}$. The network was pre-trained on the ImageNet [7] dataset.

Supplementary Figure 10 shows a similar trend as our observations on chest radiographs regarding the privacy-utility trade-off.

Supplementary Figures and Tables

	Training Set		Test Set		All	
	N	percentage	N	percentage	N	percentage
Total	153,502		39,809		193,311	
Female	52,843	(34.42%)	14,449	(36.30%)	67,292	(34.81%)
Male	100,659	(65.58%)	25,360	(63.70%)	126,019	(65.19%)
Aged [0, 30)	4,279	(2.79%)	1,165	(2.93%)	5,444	(2.82%)
Aged [30, 60)	42,340	(27.58%)	10,291	(25.85%)	52,631	(27.23%)
Aged [60, 70)	36,882	(24.03%)	10,025	(25.18%)	46,907	(24.27%)
Aged [70, 80)	48,864	(31.83%)	12,958	(32.55%)	61,822	(31.98%)
Aged [80, 100)	21,137	(13.77%)	5,370	(13.49%)	26,507	(13.71%)
Cardiomegaly	71,732	(46.72%)	18,616	(46.75%)	90,348	(46.74%)
Congestion	13,096	(8.53%)	3,275	(8.22%)	16,371	(8.47%)
Pleural effusion right	12,334	(8.03%)	3,275	(8.22%)	15,609	(8.07%)
Pleural effusion left	9,969	(6.49%)	2,602	(6.53%)	12,571	(6.50%)
Pneumonic infiltration right	17,666	(11.51%)	4,847	(12.17%)	22,513	(11.64%)
Pneumonic infiltration left	12,431	(8.10%)	3,562	(8.94%)	15,993	(8.27%)
Atelectasis right	14,841	(9.67%)	3,920	(9.84%)	18,761	(9.71%)
Atelectasis left	11,916	(7.76%)	3,166	(7.95%)	15,082	(7.80%)
	Age Training Set		Age Test Set		Age All	
	Mean	StD	Mean	StD	Mean	StD
Total	66	15	66	15	66	15
Female	66	15	66	16	66	15
Male	65	14	66	14	65	14
Aged [0, 30)	21	8	21	8	21	8
Aged [30, 60)	50	8	51	8	51	8
Aged [60, 70)	65	3	65	3	65	3
Aged [70, 80)	75	3	75	3	75	3
Aged [80, 100)	84	3	84	3	84	3

Supplementary Table 1: Statistics over subgroups of the UKA-CXR dataset used in this study. The upper part of the table shows the number of samples in each group and their relative share in training and test set, as well as the complete dataset. The lower part shows the mean and standard deviation of the age in the subgroups again over training and test sets as well as the complete dataset.

	AUROC	Accuracy	Specificity	Sensitivity
Cardiomegaly	0.84 ± 0.00	0.75 ± 0.00	0.71 ± 0.02	0.79 ± 0.02
Congestion	0.85 ± 0.00	0.75 ± 0.02	0.75 ± 0.02	0.79 ± 0.02
Pleural Effusion Right	0.94 ± 0.00	0.83 ± 0.01	0.83 ± 0.02	0.91 ± 0.02
Pleural Effusion Left	0.92 ± 0.00	0.83 ± 0.02	0.83 ± 0.02	0.86 ± 0.02
Pneumonic Infiltration Right	0.93 ± 0.00	0.85 ± 0.02	0.85 ± 0.02	0.86 ± 0.02
Pneumonic Infiltration Left	0.94 ± 0.00	0.86 ± 0.01	0.86 ± 0.02	0.87 ± 0.02
Atelectasis Right	0.89 ± 0.00	0.78 ± 0.01	0.78 ± 0.01	0.84 ± 0.02
Atelectasis Left	0.87 ± 0.00	0.78 ± 0.01	0.78 ± 0.02	0.81 ± 0.02
Average	0.90 ± 0.04	0.81 ± 0.04	0.80 ± 0.05	0.84 ± 0.04

Supplementary Table 2: Detailed evaluation results of training without DP. The results show the average and individual area under the receiver-operator-characteristic curve (AUROC), accuracy, specificity, and sensitivity values for each label tested on $N = 39,809$ test images. The training dataset includes $N = 153,502$ images.

	AUROC	Accuracy	Specificity	Sensitivity
Cardiomegaly	0.82 ± 0.00	0.73 ± 0.00	0.71 ± 0.02	0.76 ± 0.02
Congestion	0.81 ± 0.00	0.72 ± 0.02	0.71 ± 0.03	0.76 ± 0.03
Pleural Effusion Right	0.92 ± 0.00	0.82 ± 0.01	0.82 ± 0.01	0.88 ± 0.01
Pleural Effusion Left	0.89 ± 0.00	0.79 ± 0.02	0.79 ± 0.02	0.84 ± 0.02
Pneumonic Infiltration Right	0.91 ± 0.00	0.84 ± 0.01	0.83 ± 0.02	0.81 ± 0.02
Pneumonic Infiltration Left	0.91 ± 0.00	0.84 ± 0.01	0.84 ± 0.01	0.83 ± 0.01
Atelectasis Right	0.87 ± 0.00	0.78 ± 0.01	0.77 ± 0.01	0.81 ± 0.01
Atelectasis Left	0.85 ± 0.00	0.76 ± 0.02	0.76 ± 0.02	0.79 ± 0.02
Average	0.87 ± 0.04	0.79 ± 0.04	0.78 ± 0.05	0.81 ± 0.04

Supplementary Table 3: Detailed evaluation results of DP training with $\varepsilon = 7.89$, $\delta = 6 \cdot 10^{-6}$. The results show the average and individual AUROC, accuracy, specificity, and sensitivity values for each label tested on $N = 39,809$ test images. The training dataset includes $N = 153,502$ images.

	AUROC	Accuracy	Specificity	Sensitivity
Cardiomegaly	0.81 ± 0.00	0.73 ± 0.00	0.70 ± 0.01	0.77 ± 0.01
Congestion	0.81 ± 0.00	0.71 ± 0.02	0.70 ± 0.02	0.77 ± 0.02
Pleural Effusion Right	0.92 ± 0.00	0.82 ± 0.01	0.81 ± 0.01	0.87 ± 0.01
Pleural Effusion Left	0.89 ± 0.00	0.80 ± 0.01	0.80 ± 0.02	0.81 ± 0.02
Pneumonic Infiltration Right	0.90 ± 0.00	0.81 ± 0.01	0.81 ± 0.01	0.82 ± 0.01
Pneumonic Infiltration Left	0.91 ± 0.00	0.82 ± 0.01	0.82 ± 0.01	0.85 ± 0.02
Atelectasis Right	0.86 ± 0.00	0.76 ± 0.01	0.75 ± 0.02	0.83 ± 0.02
Atelectasis Left	0.85 ± 0.00	0.78 ± 0.02	0.78 ± 0.03	0.76 ± 0.03
Average	0.87 ± 0.04	0.78 ± 0.04	0.77 ± 0.05	0.81 ± 0.04

Supplementary Table 4: Detailed evaluation results of DP training with $\varepsilon = 4.71$, $\delta = 6 \cdot 10^{-6}$. The results show the average and individual AUROC, accuracy, specificity, and sensitivity values for each label tested on $N = 39,809$ test images. The training dataset includes $N = 153,502$ images.

	AUROC	Accuracy	Specificity	Sensitivity
Cardiomegaly	0.81 ± 0.00	0.73 ± 0.00	0.68 ± 0.02	0.78 ± 0.02
Congestion	0.80 ± 0.00	0.70 ± 0.02	0.69 ± 0.03	0.76 ± 0.03
Pleural Effusion Right	0.90 ± 0.00	0.80 ± 0.01	0.79 ± 0.01	0.86 ± 0.01
Pleural Effusion Left	0.87 ± 0.00	0.75 ± 0.02	0.74 ± 0.02	0.84 ± 0.02
Pneumonic Infiltration Right	0.90 ± 0.00	0.80 ± 0.01	0.80 ± 0.02	0.83 ± 0.02
Pneumonic Infiltration Left	0.90 ± 0.00	0.83 ± 0.01	0.83 ± 0.02	0.81 ± 0.02
Atelectasis Right	0.85 ± 0.00	0.74 ± 0.02	0.73 ± 0.02	0.82 ± 0.02
Atelectasis Left	0.83 ± 0.00	0.73 ± 0.03	0.73 ± 0.03	0.77 ± 0.03
Average	0.86 ± 0.04	0.76 ± 0.05	0.75 ± 0.05	0.81 ± 0.04

Supplementary Table 5: Detailed evaluation results of DP training with $\varepsilon = 2.04$, $\delta = 6 \cdot 10^{-6}$. The results show the average and individual AUROC, accuracy, specificity, and sensitivity values for each label tested on $N = 39,809$ test images. The training dataset includes $N = 153,502$ images.

	AUROC	Accuracy	Specificity	Sensitivity
Cardiomegaly	0.80 ± 0.00	0.72 ± 0.00	0.69 ± 0.02	0.76 ± 0.02
Congestion	0.80 ± 0.00	0.70 ± 0.02	0.69 ± 0.02	0.75 ± 0.02
Pleural Effusion Right	0.90 ± 0.00	0.80 ± 0.01	0.79 ± 0.02	0.86 ± 0.02
Pleural Effusion Left	0.86 ± 0.00	0.73 ± 0.02	0.72 ± 0.02	0.83 ± 0.02
Pneumonic Infiltration Right	0.89 ± 0.00	0.80 ± 0.02	0.80 ± 0.03	0.81 ± 0.03
Pneumonic Infiltration Left	0.89 ± 0.00	0.79 ± 0.01	0.79 ± 0.02	0.83 ± 0.02
Atelectasis Right	0.84 ± 0.00	0.74 ± 0.02	0.74 ± 0.02	0.80 ± 0.02
Atelectasis Left	0.82 ± 0.00	0.70 ± 0.01	0.69 ± 0.02	0.79 ± 0.02
Average	0.85 ± 0.04	0.75 ± 0.04	0.74 ± 0.05	0.80 ± 0.04

Supplementary Table 6: Detailed evaluation results of DP training with $\varepsilon = 1.06$, $\delta = 6 \cdot 10^{-6}$. The results show the average and individual AUROC, accuracy, specificity, and sensitivity values for each label tested on $N = 39,809$ test images. The training dataset includes $N = 153,502$ images.

	AUROC	Accuracy	Specificity	Sensitivity
Cardiomegaly	0.79 ± 0.00	0.72 ± 0.00	0.69 ± 0.01	0.74 ± 0.01
Congestion	0.79 ± 0.00	0.67 ± 0.02	0.66 ± 0.02	0.78 ± 0.02
Pleural Effusion Right	0.89 ± 0.00	0.77 ± 0.01	0.76 ± 0.02	0.86 ± 0.02
Pleural Effusion Left	0.84 ± 0.00	0.71 ± 0.02	0.70 ± 0.03	0.84 ± 0.03
Pneumonic Infiltration Right	0.88 ± 0.00	0.80 ± 0.01	0.80 ± 0.02	0.79 ± 0.02
Pneumonic Infiltration Left	0.88 ± 0.00	0.77 ± 0.02	0.77 ± 0.03	0.83 ± 0.03
Atelectasis Right	0.83 ± 0.00	0.74 ± 0.01	0.73 ± 0.01	0.79 ± 0.01
Atelectasis Left	0.81 ± 0.00	0.70 ± 0.03	0.70 ± 0.03	0.77 ± 0.03
Average	0.84 ± 0.04	0.73 ± 0.04	0.73 ± 0.05	0.80 ± 0.04

Supplementary Table 7: Detailed evaluation results of DP training with $\varepsilon = 0.54$, $\delta = 6 \cdot 10^{-6}$. The results show the average and individual AUROC, accuracy, specificity, and sensitivity values for each label tested on $N = 39,809$ test images. The training dataset includes $N = 153,502$ images.

	AUROC	Accuracy	Specificity	Sensitivity
Cardiomegaly	0.79 ± 0.00	0.71 ± 0.00	0.67 ± 0.01	0.75 ± 0.01
Congestion	0.78 ± 0.00	0.68 ± 0.02	0.68 ± 0.02	0.74 ± 0.02
Pleural Effusion Right	0.88 ± 0.00	0.77 ± 0.01	0.77 ± 0.02	0.83 ± 0.02
Pleural Effusion Left	0.84 ± 0.00	0.73 ± 0.01	0.72 ± 0.02	0.80 ± 0.02
Pneumonic Infiltration Right	0.87 ± 0.00	0.79 ± 0.01	0.79 ± 0.02	0.79 ± 0.02
Pneumonic Infiltration Left	0.88 ± 0.00	0.79 ± 0.01	0.79 ± 0.01	0.81 ± 0.01
Atelectasis Right	0.82 ± 0.00	0.73 ± 0.02	0.73 ± 0.02	0.77 ± 0.02
Atelectasis Left	0.80 ± 0.00	0.71 ± 0.02	0.71 ± 0.02	0.75 ± 0.02
Average	0.83 ± 0.04	0.74 ± 0.04	0.73 ± 0.05	0.78 ± 0.04

Supplementary Table 8: Detailed evaluation results of DP training with $\varepsilon = 0.29$, $\delta = 6 \cdot 10^{-6}$. The results show the average and individual AUROC, accuracy, specificity, and sensitivity values for each label tested on $N = 39,809$ test images. The training dataset includes $N = 153,502$ images.

	PDAC													
	Total		Male		Female		Youngest 25%		Second 25%		Third 25%		Oldest 25%	
ε	μ	σ	μ	σ	μ	σ	μ	σ	μ	σ	μ	σ	μ	σ
0.29	24.86	10.7	23.86	9.6	25.54	14.0	20.29	23.9	15.97	7.9	32.10	8.8	27.78	12.0
0.54	11.37	3.2	11.23	3.4	10.82	4.2	8.70	8.7	4.86	2.4	19.14	7.0	10.42	2.1
1.06	5.97	1.7	5.96	1.6	6.06	2.0	2.90	2.5	1.39	2.4	11.11	3.7	6.25	2.1
2.04	2.70	0.9	2.46	0.6	3.03	1.5	1.45	2.5	1.39	1.2	3.09	1.1	4.17	3.6
4.71	1.73	1.0	1.40	0.6	2.16	1.5	1.45	2.5	0.69	1.2	1.85	0.0	2.78	1.2
5.0	2.31	2.0	1.75	1.2	3.03	3.0	1.45	2.5	1.39	2.4	2.47	1.1	3.47	2.4
6.0	3.08	2.3	2.46	2.2	3.90	2.6	1.45	2.5	2.08	2.1	3.70	3.2	4.17	2.1
7.0	1.54	1.2	1.40	1.6	1.73	1.5	0.00	0.0	0.69	1.2	2.47	2.8	2.08	2.1
8.0	0.58	0.6	0.00	0.0	1.30	1.3	0.00	0.0	1.39	2.4	0.00	0.0	0.69	1.2
Non-private	0.77	0.7	0.00	0.0	1.73	1.5	0.00	0.0	2.08	2.1	0.62	1.1	0.00	0.0

Supplementary Table 9: Underdiagnosis rates of subgroups. Underdiagnosis rate is the false positive rate of non-tumor cases. μ denotes the mean underdiagnosis rate for a certain subgroup, while σ denotes the standard deviation.

	Tumor		Control		PtD	
N Test	173		152			
ε	μ	σ	μ	σ	μ	σ
0.29	75.14	10.7	85.09	2.3	−9.94	13.0
0.54	88.63	3.2	86.40	2.5	2.23	5.4
1.06	94.03	1.7	85.53	3.5	8.50	4.7
2.04	97.30	0.9	87.94	1.0	9.36	0.4
4.71	98.27	1.0	90.57	1.9	7.70	2.9
5.0	97.69	2.0	91.01	2.1	6.68	4.1
6.0	96.92	2.3	91.89	1.7	5.03	4.0
7.0	98.46	1.2	90.79	1.7	7.67	2.8
8.0	99.42	0.6	95.39	3.7	4.03	3.5
∞	99.23	0.7	97.81	1.5	1.42	1.3

Supplementary Table 10: Per Diagnosis Accuracy on the PDAC dataset. PtD is the statistical parity difference between the tumor and control group. μ denotes the mean, σ the standard deviation over three runs.

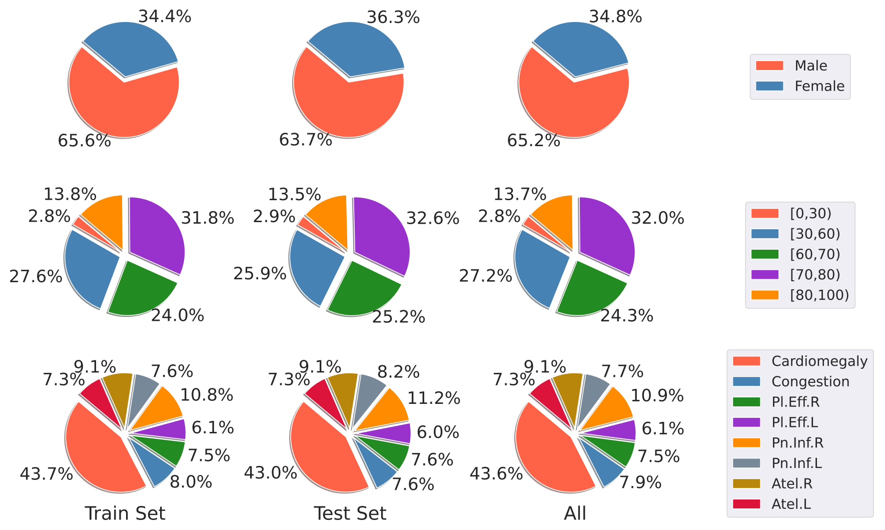

(a) UKA-CXR dataset

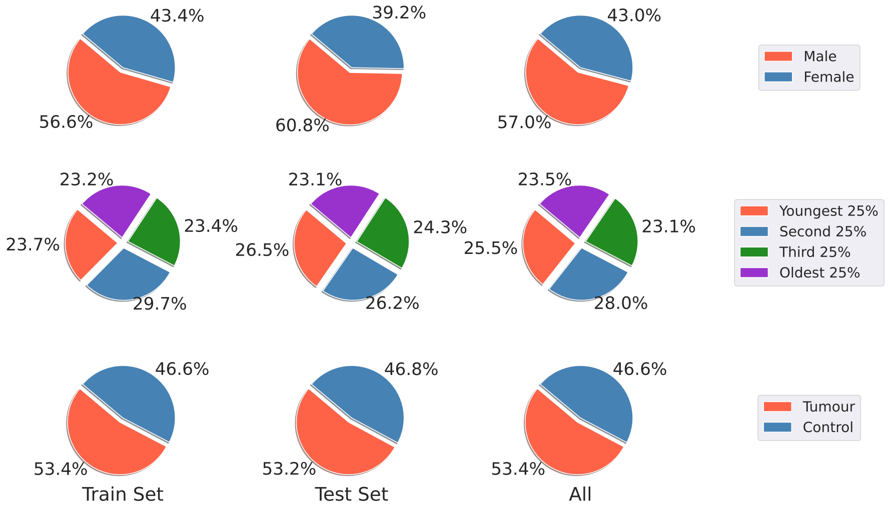

(b) PDAC dataset

Supplementary Figure 1: Visual overview of the distribution over subgroups

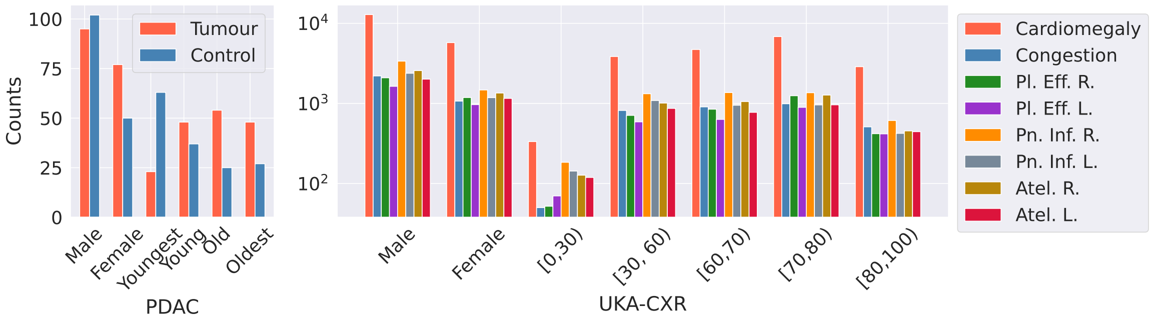

Supplementary Figure 2: Distribution of labels within subgroups

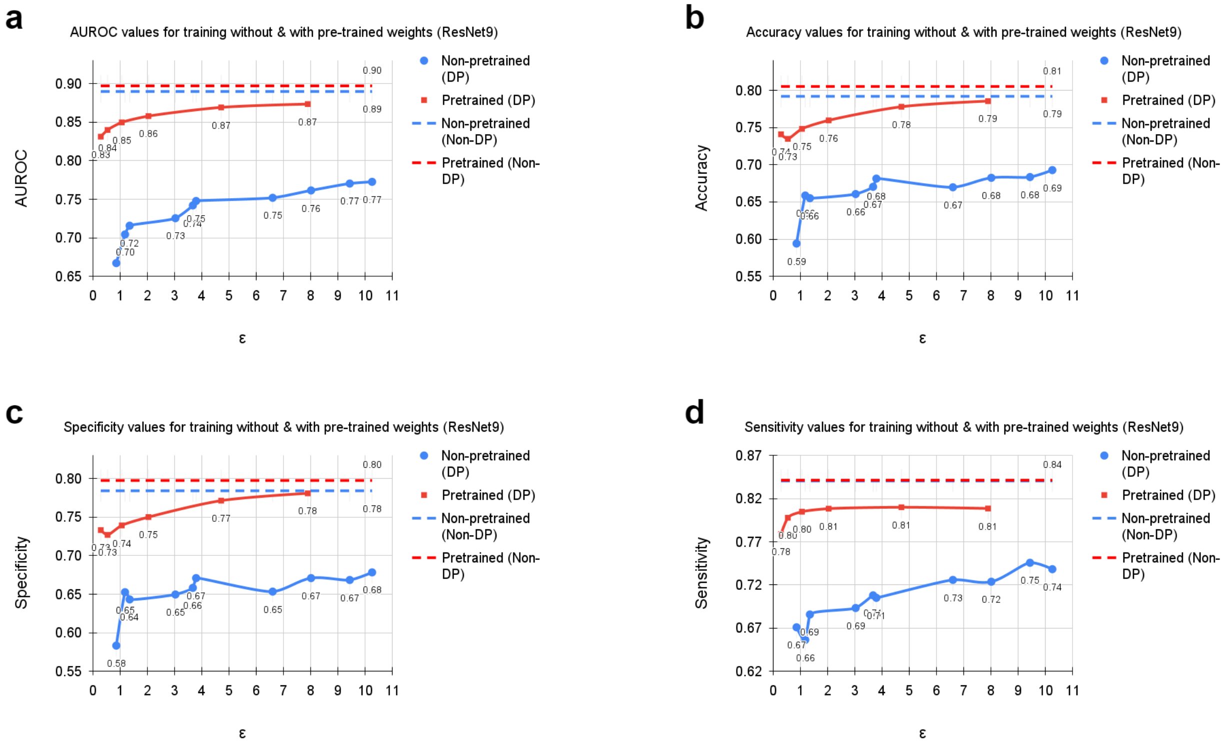

Supplementary Figure 3: Average results of DP training with different ε values for $\delta = 6 \cdot 10^{-6}$ using pre-trained weights versus training from scratch. The curves show the average **a** AUROC, **b** accuracy, **c** specificity, and **d** sensitivity values over all labels, including cardiomegaly, congestion, pleural effusion right, pleural effusion left, pneumonic infiltration right, pneumonic infiltration left, atelectasis right, and atelectasis left tested on $N = 39\,809$ test images. The training dataset includes $N = 153\,502$ images. Note, that the AUROC is monotonically increasing, while sensitivity, specificity, and accuracy exhibit more variation. This is due to the fact that all training processes were optimized for the AUROC. Dashed lines correspond to the non-private training results depicted as upper bounds. The pre-training was done using the MIMIC-CXR dataset with $N = 210\,652$ images.

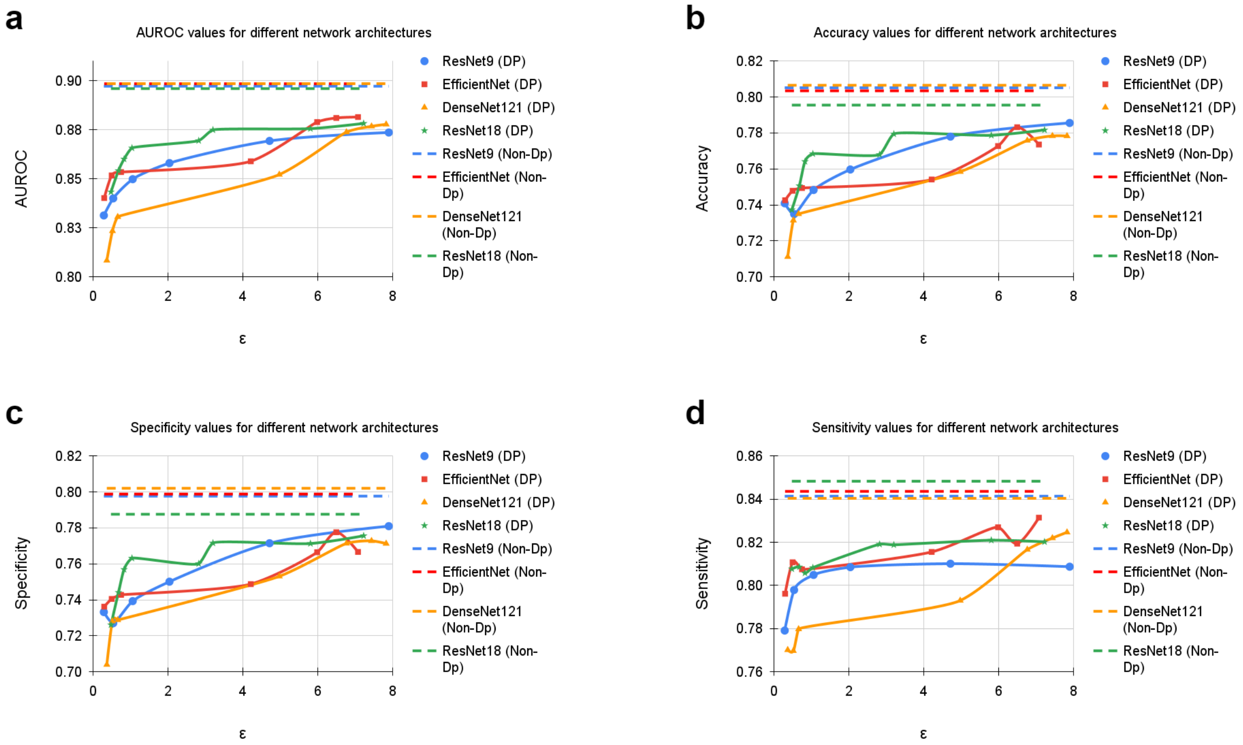

Supplementary Figure 4: Average results of training with DP with different ε values for $\delta = 6 \cdot 10^{-6}$ using different network architectures. The curves show the average **a** AUROC, **b** accuracy, **c** specificity, and **d** sensitivity values over all labels, including cardiomegaly, congestion, pleural effusion right, pleural effusion left, pneumonic infiltration right, pneumonic infiltration left, atelectasis right, and atelectasis left tested on $N = 39\,809$ test images. The training dataset includes $N = 153\,502$ images. Note, that the AUROC is monotonically increasing, while sensitivity, specificity, and accuracy exhibit more variation. This is due to the fact that all training processes were optimized for the AUROC. Dashed lines correspond to the non-private training results depicted as upper bounds.

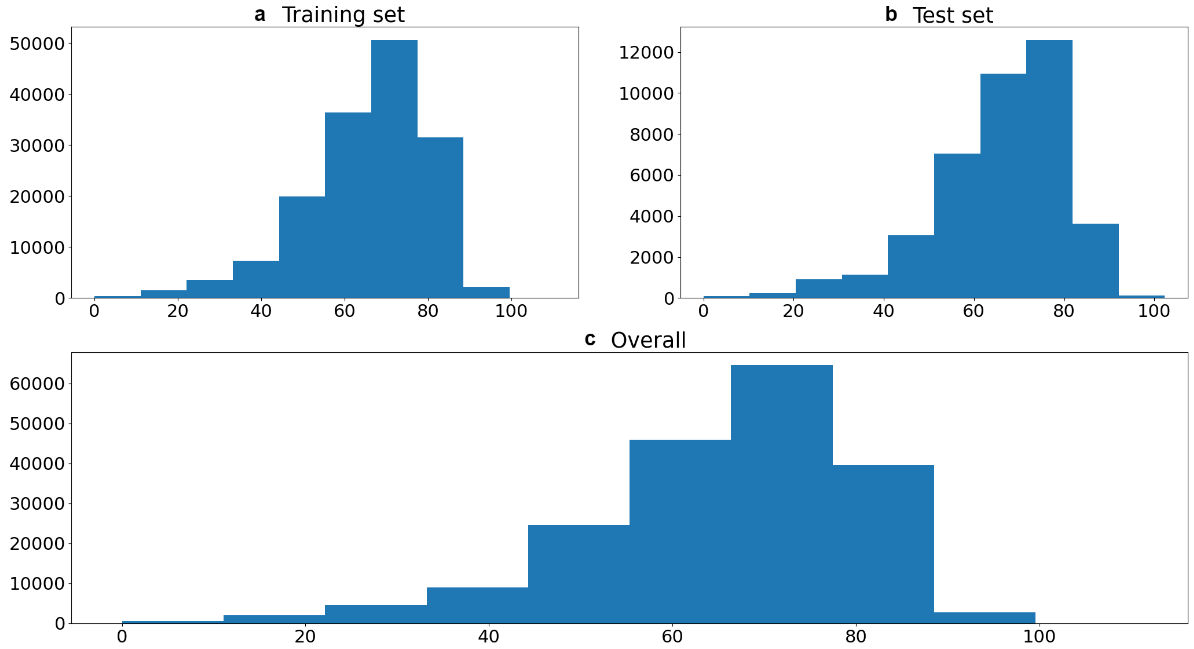

Supplementary Figure 5: Age histogram of the UKA-CXR dataset. **a** Training set. **b** Test set. **c** Overall.

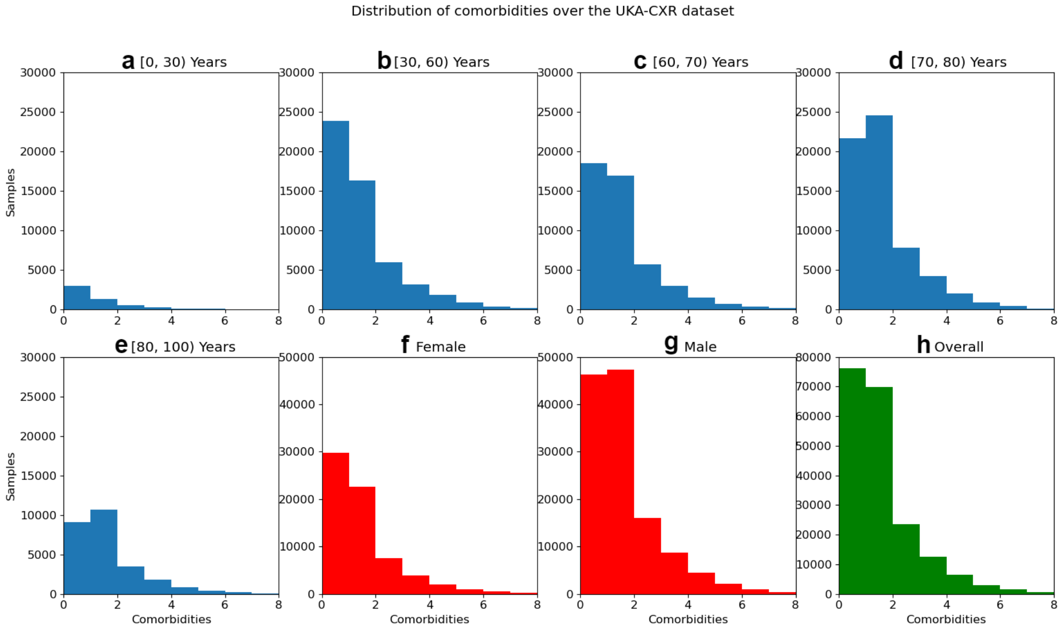

Supplementary Figure 6: Distribution of comorbidities over the UKA-CXR dataset. Histograms of comorbidities are given for different subsets of the dataset including subjects aging in the range of **a** $[0, 30)$ years old with a mean of 0.8 ± 1.2 comorbidities, **b** $[30, 60)$ years old with a mean of 1.0 ± 1.3 comorbidities, **c** $[60, 70)$ years old with a mean of 1.1 ± 1.3 comorbidities, **d** $[70, 80)$ years old with a mean of 1.1 ± 1.2 comorbidities, **e** $[80, 100)$ years old with a mean of 1.1 ± 1.3 comorbidities, as well as **f** females with a mean of 1.0 ± 1.2 comorbidities, **g** males with a mean of 1.1 ± 1.3 comorbidities, and **h** overall with a mean of 1.1 ± 1.3 comorbidities.

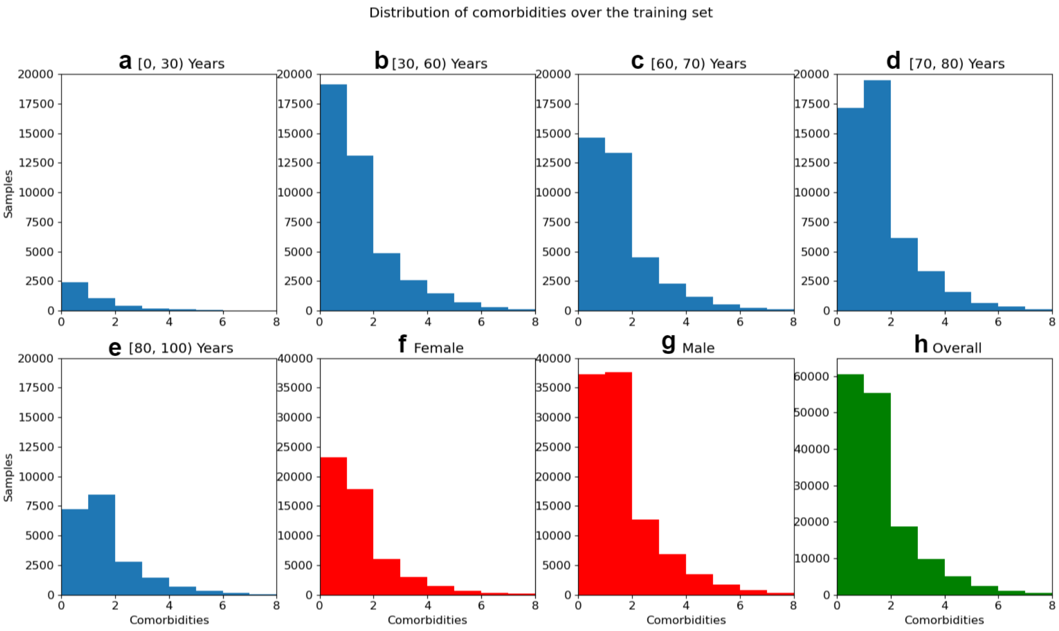

Supplementary Figure 7: Distribution of comorbidities over the training set. Histograms of comorbidities are given for different subsets of the training set including subjects aging in the range of **a** $[0, 30)$ years old with a mean of 0.8 ± 1.2 comorbidities, **b** $[30, 60)$ years old with a mean of 1.0 ± 1.3 comorbidities, **c** $[60, 70)$ years old with a mean of 1.1 ± 1.3 comorbidities, **d** $[70, 80)$ years old with a mean of 1.1 ± 1.2 comorbidities, **e** $[80, 100)$ years old with a mean of 1.1 ± 1.3 comorbidities, as well as **f** females with a mean of 1.0 ± 1.2 comorbidities, **g** males with a mean of 1.1 ± 1.3 comorbidities, and **h** overall training set with a mean of 1.1 ± 1.3 comorbidities.

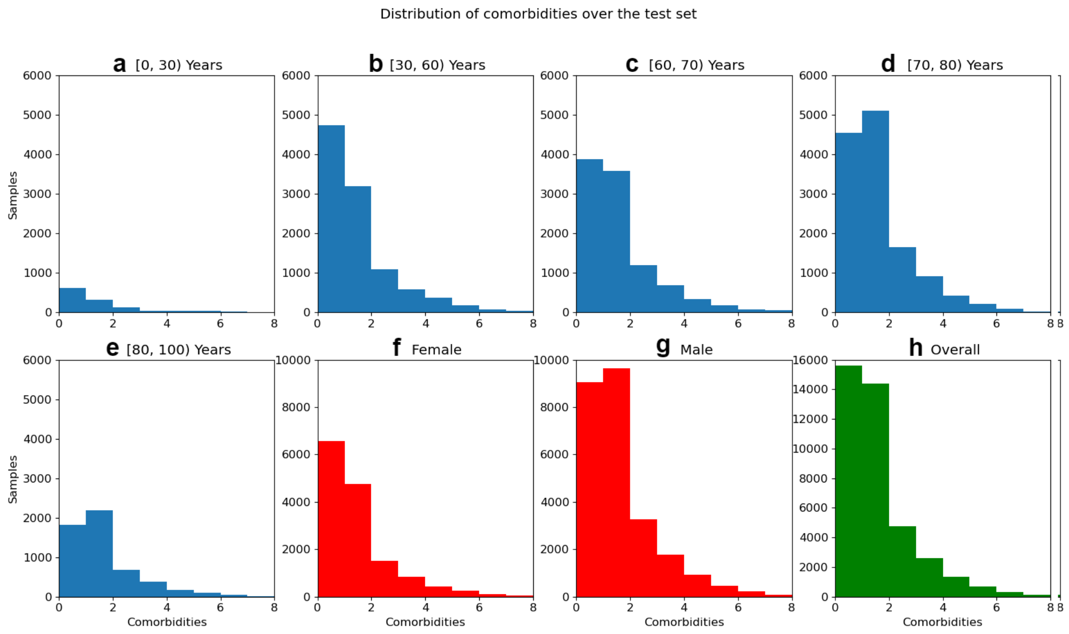

Supplementary Figure 8: Distribution of comorbidities over the test set. Histograms of comorbidities are given for different subsets of the test set including subjects aging in the range of **a** $[0, 30)$ years old with a mean of 0.9 ± 1.4 comorbidities, **b** $[30, 60)$ years old with a mean of 1.0 ± 1.3 comorbidities, **c** $[60, 70)$ years old with a mean of 1.1 ± 1.3 comorbidities, **d** $[70, 80)$ years old with a mean of 1.1 ± 1.2 comorbidities, **e** $[80, 100)$ years old with a mean of 1.1 ± 1.3 comorbidities, as well as **f** females with a mean of 1.0 ± 1.3 comorbidities, **g** males with a mean of 1.1 ± 1.3 comorbidities, and **h** overall test set with a mean of 1.1 ± 1.3 comorbidities.

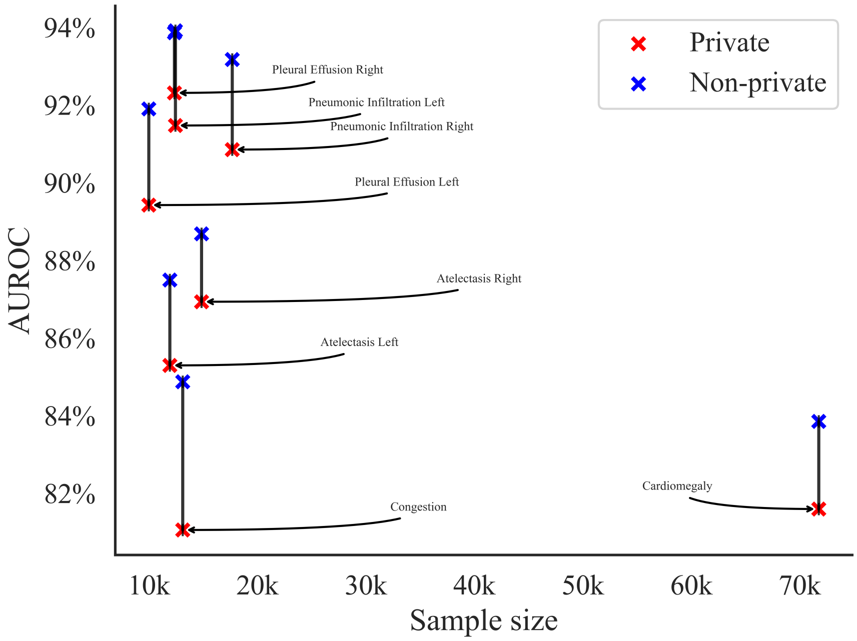

Supplementary Figure 9: Relation of sample size to training performance for private and performance loss compared to non private training. Each dot marks the performance on the test set on one diagnosis of the private model at $\varepsilon = 7.89$. Colors indicate the performance loss compared to the non private model.

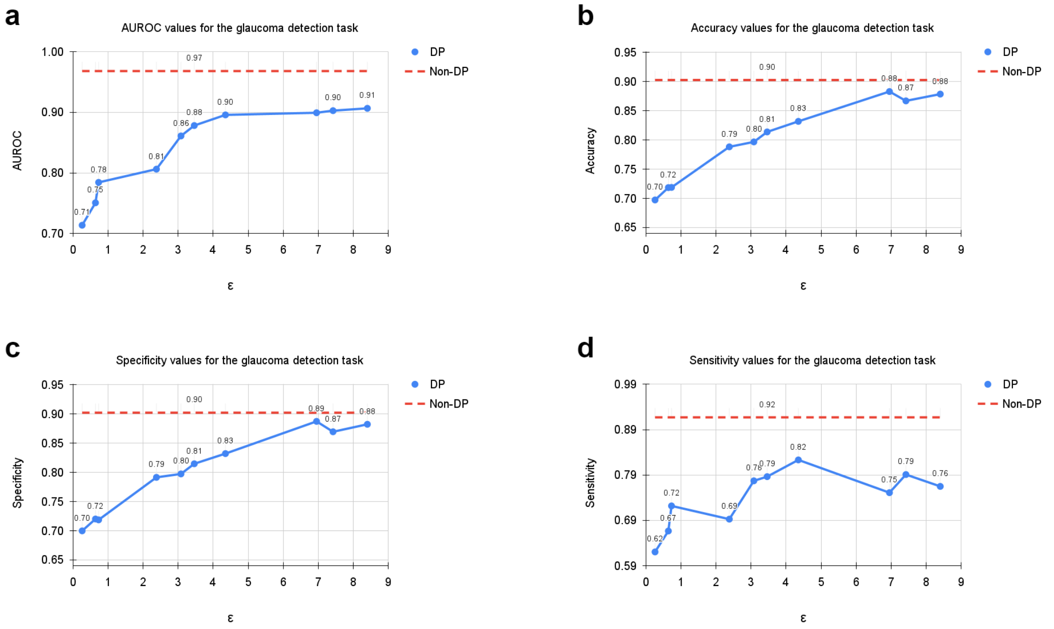

Supplementary Figure 10: Evaluation results of the Glaucoma detection task [4] for training with DP with different ε values for $\delta = 6 \cdot 10^{-6}$. The curves show the **a** AUROC, **b** accuracy, **c** specificity, and **d** sensitivity values tested on $N = 20\,268$ test images. The training dataset includes $N = 81\,086$ images. Note, that the AUROC is monotonically increasing, while sensitivity, specificity, and accuracy exhibit more variation. This is due to the fact that all training processes were optimized for the AUROC. Dashed lines correspond to the non-private training results depicted as upper bounds.

Supplementary References

[1] Mingxing Tan and Quoc Le. Efficientnet: Rethinking model scaling for convolutional neural networks. In *Proceedings of the 36th International Conference on Machine Learning*, pages 6105–6114, 2019.

[2] Gao Huang, Zhuang Liu, Laurens van der Maaten, and Kilian Q. Weinberger. Densely connected convolutional networks. In *Proceedings of the IEEE Conference on Computer Vision and Pattern Recognition (CVPR)*, pages 4700–4708, 2017.

[3] Kaiming He, Xiangyu Zhang, Shaoqing Ren, and Jian Sun. Deep residual learning for image recognition. In *Proceedings of the IEEE conference on computer vision and pattern recognition*, pages 770–778, 2016.

[4] Coen de Vente, Koenraad A. Vermeer, Nicolas Jaccard, He Wang, Hongyi Sun, Firas Khader, Daniel Truhn, Temirgali Aimyshev, Yerkebulan Zhanibekuly, Tien-Dung Le, Adrian Galdran, Miguel Ángel González Ballester, Gustavo Carneiro, Devika R G, Hrishikesh P S, Densen Puthussery, Hong Liu, Zekang Yang, Satoshi Kondo, Satoshi Kasai, Edward Wang, Ashritha Durvasula, Jónathan Heras, Miguel Ángel Zapata, Teresa Araújo, Guilherme Aresta, Hrvoje Bogunović, Mustafa Arikan, Yeong Chan Lee, Hyun Bin Cho, Yoon Ho Choi, Abdul Qayyum, Imran Razzak, Bram van Ginneken, Hans G. Lemij, and Clara I. Sánchez. Airogs: Artificial intelligence for robust glaucoma screening challenge. *arXiv preprint arXiv:2302.01738*, 2023.

[5] Firas Khader, Christoph Haarburger, Jörg-Christian Kirr, Marcel Menke, Jakob Nikolas Kather, Johannes Stegmaier, Christiane Kuhl, Sven Nebelung, and Daniel Truhn. Elevating fundoscopic evaluation to expert level - automatic glaucoma detection using data from the airogs challenge. In *2022 IEEE International Symposium on Biomedical Imaging Challenges (ISBIC)*, pages 1–4, 2022.

[6] Ahmed Al-Mahrooqi, Dmitrii Medvedev, Rand Muhtaseb, and Mohammad Yaqub. Gardnet: Robust multi-view network for glaucoma classification in color fundus images. In Bhavna Antony, Huazhu Fu, Cecilia S. Lee, Tom MacGillivray, Yanwu Xu, and Yalin Zheng, editors, *Ophthalmic Medical Image Analysis*, pages 152–161, Cham, 2022. Springer International Publishing.

[7] Jia Deng, Wei Dong, Richard Socher, Li-Jia Li, Kai Li, and Li Fei-Fei. Imagenet: A large-scale hierarchical image database. In *2009 IEEE Conference on Computer Vision and Pattern Recognition*, pages 248–255, 2009.
