# Supplementary material for: Preserving fairness and diagnostic accuracy in private large-scale AI models for medical imaging
Source: Commun Med (Lond). 2024 Mar 14;4:46. doi: 10.1038/s43856-024-00462-6 (PMC10940659; doi:10.1038/s43856-024-00462-6)
Supplement: Supplementary file 3 — Description of Additional Supplementary Files [file 43856_2024_462_MOESM3_ESM.pdf]

Description of Additional Supplementary Files

File Name: Supplementary Data 1
Description: Source data
